# Effects of Subauroral Polarization Streams on the Upper Thermospheric Winds During Non‐Storm Time

**DOI:** 10.1029/2021JA029988

**Published:** 2022-04-27

**Authors:** Ying Zou, Larry R. Lyons, Xueling Shi, Jiang Liu, Qian Wu, Mark Conde, Simon G. Shepherd, Stephen Mende, Yongliang Zhang, Antea Coster

**Affiliations:** ^1^ Department of Space Science University of Alabama in Huntsville Huntsville AL USA; ^2^ Department of Atmospheric and Oceanic Sciences University of California, Los Angeles Los Angeles CA USA; ^3^ The Bradley Department of Electrical and Computer Engineering Virginia Tech Blacksburg VA USA; ^4^ High Altitude Observatory National Center for Atmospheric Research Boulder CO USA; ^5^ Department of Earth, Planetary and Space Sciences University of California, Los Angeles Los Angeles CA USA; ^6^ Department of Physics University of Alaska Fairbanks Fairbanks AK USA; ^7^ Thayer School of Engineering Dartmouth College Hanover NH USA; ^8^ Space Sciences Laboratory University of California Berkeley CA USA; ^9^ Applied Physics Laboratory Laurel MD USA; ^10^ Massachusetts Institute of Technology Haystack Observatory Westford MA USA

**Keywords:** SAPS, upper thermosphere, neutral wind, ion‐neutral coupling

## Abstract

Intense sunward (westward) plasma flows, named Subauroral Polarization Stream (SAPS), have been known to occur equatorward of the electron auroras for decades, yet their effect on the upper thermosphere has not been well understood. On the one hand, the large velocity of SAPS results in large momentum exchange upon each ion‐neutral collision. On the other hand, the low plasma density associated with SAPS implies a low ion‐neutral collision frequency. We investigate the SAPS effect during non‐storm time by utilizing a Scanning Doppler Imager (SDI) for monitoring the upper thermosphere, SuperDARN radars for SAPS, all‐sky imagers and DMSP Spectrographic Imager for the auroral oval, and GPS receivers for the total electron content. Our observations suggest that SAPS at times drives substantial (>50 m/s) westward winds at subauroral latitudes in the dusk‐midnight sector, but not always. The occurrence of the westward winds varies with *AE* index, plasma content in the trough, and local time. The latitudinally averaged wind speed varies from 60 to 160 m/s, and is statistically 21% of the plasma. These westward winds also shift to lower latitude with increasing *AE* and increasing MLT. We do not observe SAPS driving poleward wind surges, neutral temperature enhancements, or acoustic‐gravity waves, likely due to the somewhat weak forcing of SAPS during the non‐storm time.

## Key Points

- Subauroral Polarization Stream (SAPS) at times drives substantial (>; 50‐m/s) westward winds at subauroral latitudes in the dusk‐midnight sector, but not always
- The occurrence of the SAPS‐driven winds varies with AE index, plasma content in the trough, and the local time
- The wind speed is 21% of SAPS flow, implying a weaker ion‐neutral coupling during non‐storm than active time

## 1 Introduction

Thermospheric wind circulation is of crucial importance because it fundamentally influences the composition and dynamics of the neutral and ionized components of the upper atmosphere. At high latitudes, winds are driven primarily by the momentum exchange between the convecting ions and the neutral gas, pressure gradients produced by the dayside solar heating, joule heating, and auroral particle precipitation, and inertial forces (e.g., Coriolis and centrifugal) (e.g., Killeen & Roble, 1988; Kwak & Richmond, 2007; Meriwether et al., 1973; Mikkelsen & Larsen, 1983; Richmond et al., 2003; Roble et al., 1982; Thayer & Killeen, 1993). Under a southward IMF and in the polar cap, ions convect in a direction similar to the pressure gradient produced by the dayside solar heating, both directed anti‐sunward, and winds in the upper thermosphere flow anti‐sunward across the pole. In the auroral zone, ions convect oppositely to the pressure gradient, and winds show a semidiurnal variation, i.e., directed sunward in the nightside sector, and anti‐sunward in the dayside. Both the polar cap and auroral zone winds vary with season, solar activity, IMF, and geomagnetic activity (e.g., Aruliah et al., 1996; Aruliah, Rees, & Fuller‐Rowell, 1991; Aruliah, Rees, & Steen, 1991; Dhadly et al., 2017, 2018; Emmert et al., 2006; Förster et al., 2008; Fuller‐Rowell et al., 1996; Killeen, 1987; Killeen et al., 1995; McCormac et al., 1985, 1987, 1991; McCormac & Smith, 1984; Niciejewski et al., 1992; Rees et al., 1989; Sica et al., 1989; Witasse et al., 1998, 2008; Wu et al., 2004).

However, much less attention has been paid to the wind circulation at subauroral latitudes, despite that the ions there can drift at a high speed of several hundreds to more than 1,000 m/s during subauroral polarization stream (SAPS) events. SAPS is intense sunward (westward) plasma flows driven by large poleward electric fields in the subauroral ionosphere equatorward of the electron auroras (Foster & Burke, 2002). The phenomenon encompasses latitudinally broad regions (3°–5° wide) of flows often exceeding hundreds of m/s (Erickson et al., 2011; Foster & Vo, 2002), and latitudinally narrow channels (1°–2°) of flows often exceeding 1 km/s. The latter is known as polarization jets (Galperin et al., 1974) or subauroral ion drifts (SAID) (Anderson et al., 1993, 2001; Spiro et al., 1979). SAPS may also comprise quasiperiodic electromagnetic wave structures at scale sizes of tens of kilometers in the ionosphere (Makarevich & Bristow, 2014; Mishin & Burke, 2005; Mishin et al., 2002, 2003). As suggested by several climatological studies (Erickson et al., 2011; Foster & Vo, 2002; Kunduri et al., 2017; Landry & Anderson, 2018), SAPS often occurs at 60°–65° MLAT in the dusk sector. The magnitude of the velocity increases with increasing geomagnetic activity and towards dusk. The latitude of the peak velocity decreases linearly with increasing geomagnetic activity and towards midnight. SAPS is localized to the midnight sector during quiet time, and extends to earlier MLT for increasing geomagnetic activity. SAPS is usually associated with a midlatitude trough of 15%–20% depletion in the background density (Aa et al., 2020).

Since discovery, SAPS has been recognized to play an important role in redistributing plasma in the geospace system thanks to its contribution to enhanced ion vertical flows (e.g., Wang & Lühr, 2013), storm‐enhanced density plumes (e.g., Foster et al., 2007; Zou et al., 2014), and sunward‐convecting plasmaspheric drainage plumes (e.g., Goldstein et al., 2005). However, its effect on thermospheric neutrals has not been well understood. On one hand, the large velocity of SAPS results in large momentum exchange upon each collision between the convecting ions and the neutrals. On the other hand, the low plasma density associated with SAPS implies a low ion‐neutral collision frequency. Whether and how SAPS affects the thermosphere at subauroral latitudes is the science question we address in this study.

Existing studies on the effect of SAPS are limited in numbers, and most of them are further limited to strong SAPS events. H. Wang et al. (2011) reported that, based on coordinated CHAMP and DMSP observations, strong sunward winds occur in association with SAPS and show a peak at the same latitude as the SAPS flow. The peak wind velocity is on the order of 200 m/s and is 35% of that of the plasma. The thermospheric mass density is also enhanced by about 10% at 400 km altitude, likely due to frictional heating. By studying winds at various local time sectors relative to SAPS, Wang, Lühr, et al. (2012) suggested that SAPS has the strongest effect at 19 h MLT, and relatively little effect around 23–24 h MLT. However, the SAPS events studied in Wang, Lühr, et al. (2012) are overall wider in latitude (5°–10° wide) than normal. Whether the normal SAPS (usually 3°–5° wide (Foster & Vo, 2002)) can drive sizeable westward winds remains unclear. In another observational study, Zhang et al. (2015) examined SAPS during a super geomagnetic storm using joint observations of an incoherent scatter radar and Fabry‐Perot interferometers. They found that the storm‐time SAPS drives a westward wind of ∼300 m/s amplitude. This westward wind was deflected due to the Coriolis effect, forming a poleward wind surge of 100 m/s in a few hours following the onset of SAPS.

Through simulation, Wang, Talaat, et al. (2012) concluded that SAPS accelerates thermospheric winds. The zonal winds have either an extra, separate channel of westward flow in the subauroral region, or a broad westward wind jet that merges with the winds in the auroral zone. SAPS can also cause an increase in global thermospheric temperature. The peak temperature enhancement is as large as about 60 K and occurs around the SAPS channel. Also based on simulation, Guo et al. (2018) revealed that SAPS drives, in addition to the westward wind channel, a pair of wind vortices along its northern (clockwise) and southern (anticlockwise) edges. They further found that SAPS serves as an important source of traveling atmospheric waves, and proposed that the poleward wind surge detected by Zhang et al. (2015) was more contributed by these waves than the Coriolis effect. Ferdousi et al. (2019) examined forces that drive the SAPS‐induced winds and found that ion drag is the dominant force.

In this paper we study the effect of non‐storm SAPS on the upper thermosphere when the SAPS driving is modest or weak, and compare the observations with model predictions. Section 2 describes the utilized instruments and our event selection criteria. Section 3 displays three case studies where SAPS affects the thermosphere to different degrees. Section 4 explores the factors controlling the strength of the SAPS effect and Section 5 provides the summary of the study.

## 2 Methodology

Observations of the upper thermosphere are obtained by the Scanning Doppler Imager (SDI) located at High‐frequency Active Auroral Research Program (HRP, geographic 62.4°N, −145.1°E, geomagnetic 62.9°N, −87.9°E, operated from October 2009 to May 2014). We focus on this SDI because it has a field‐of‐view (FOV) covering 58°–70° in MLAT, where SAPS tends to occur (Erickson et al., 2011; Foster & Vo, 2002). The basic principles behind, and the operations of, SDIs are described in detail in Conde and Smith (1995, 1998), Conde and Nicolls (2010), and Dhadly et al. (2015), and are briefly reviewed here. SDIs collect optical emissions simultaneously from a wide FOV (typically 140°). The emissions are passed through a narrowband interference filter and then a separation‐scanned etalon, from which the emission spectra are resolved. The Doppler shift of the emission contains information of line‐of‐sight (LOS) wind speeds, and the Doppler broadening contains temperatures. The emission used in the current study is the 630.0 nm emissions originated from the upper thermosphere (presumably at 250 km). Although the exact emission profile may change with auroral precipitation, upper‐thermospheric winds are known to only vary gradually with altitudes (Deng & Ridley, 2006, 2008; Wang et al., 2008). The temporal resolution of the wind measurements is 1–5 min, and the spatial resolution is ∼0.3° in latitude near the zenith and ∼1° near the FOV edge.

SDI LOS velocities are inverted to horizontal velocity vectors by applying a monostatic fitting algorithm (Conde & Smith, 1998). This algorithm assumes that vertical winds are constant across the FOV and that meridional winds are stationary in local time. These assumptions haven been found to be reasonable even for rapidly evolving and/or strongly sheared winds (Anderson et al., 2012; Dhadly et al., 2015). We further check the fidelity of the fitted wind vectors by comparing the vectors fitted of HRP SDI with those of the SDI located at Poker Flat (PKR, geographic 65.1°N, −147.4°E, geomagnetic 65.5°N, −91.4°E) whenever data are available. The fittings from the two stations give very similar results, except that at times a small offset of low tens of m/s can exist (see Zou et al. (2021) and results in Section 3.1).

To identify SAPS, accurate information of the equatorward boundary of the auroral oval is necessary. The boundary is primarily determined using the THEMIS all‐sky imager (ASI) located at Gakona, i.e., the same location as the HRP SDI. This boundary is further validated by DMSP SSUSI observations at the LBH short (LBHS) wavelength. SSUSI observations provide maps of the auroral emissions along the spacecraft trajectory every 100 min.

To obtain plasma convection equatorward of the auroral oval, SuperDARN radars located at King Salmon (KSR, geographic 58.7°N, −156.7°E, geomagnetic 57.5°N, −99.1°E) and Christmas Valley West (CVW, geographic 43.3°N, −120.4°E, geomagnetic 49.5°N, −58.3°E) are employed because they capture the convection neighboring the SDI wind vectors. Although the radar located at Adak Island East (ADE, geographic 51.9°N, −176.6°E, geomagnetic 47.6°N, −113.0°E) also captures the nearby convection, we found no measurements from this radar satisfying the event selection criteria detailed below.

When surveying events, we require the sky condition to be good at Gakona, which ensures that the SDI wind vectors and the equatorward boundary of the auroral oval are reliable. A good sky condition means that auroral forms are clearly discernible in the THEMIS ASI data. The equatorward boundary of the auroral oval should be located at >62° MLAT so that SAPS is largely located within the SDI FOV. This requirement effectively excludes geomagnetic storms, because during storms the equatorward boundary of the oval extends further equatorward. In fact, the Kp index of all our events is <4.

We also require that events are associated with good SuperDARN backscatter echoes equatorward of the auroral oval for >2 h, and SAPS events are identified as when the radar LOS velocities are >200 m/s moving sunward within the dusk‐midnight sector. Our focus is on the relatively broad SAPS rather than SAID, i.e., extending ≥2° from the auroral equatorward boundary, so that its effect on the thermosphere can be sufficiently resolved by the SDI. SAPS can occur outside the Alaska region, but due to the lack of thermospheric wind measurements, those events are beyond the scope of the study. For interested readers, SAPS evolution on a global scale is presented in Figure S1 of Supporting Information S1 using SuperDARN global convection maps. SAPS is regarded to have a significant effect on thermospheric wind circulation if it drives westward winds with a speed >50 m/s at subauroral latitudes (uncertainties of wind vectors are 15–20 m/s (Anderson et al., 2012; Dhadly et al., 2015)). Note that H. Wang et al. (2011) suggested that the wind's velocity is usually 35% of the plasma at SAPS. Therefore, our threshold of 50 m/s is low enough to include typical winds associated with SAPS of >200 m/s, and at the same time significantly larger than the measurement uncertainty so that the wind behavior is trustworthy. Winds slower than 50 m/s may still reflect SAPS forcing, but the small velocity implies a weak effect.

We additionally examine the plasma content at SAPS by using the GPS total electron content (TEC) obtained from the Madrigal database maintained by Massachusetts Institute of Technology, Haystack Observatory (Rideout & Coster, 2006). One TEC unit (TECU) is given as 1 × 10^16^ el/m^2^ and represents the total number of electrons contained in a column extending upward from the Earth's surface through the ionosphere with a cross‐sectional area of 1 m^2^. Because the F region of the ionosphere is the main contributor to the electron content, TEC is used as a proxy to characterize the state of the F region.

We have identified 14 SAPS events satisfying the above criteria between October 2009 and May 2014, and examine whether and how SAPS affects the upper thermospheric winds. Three representative events with the best data coverage of SAPS are selected and presented as case studies in the following section. Table 1 lists the intervals of all the 14 events and the maximum AE and Kp index during the intervals. The statistical properties are presented in Section 4.

**Table 1:** Intervals of the Studied SAPS Events That Have Simultaneous Auroral, Neutral Wind, and Plasma Convection Measurements

Intervals	Max AE	UT at Max AE	Max Kp	UT at max Kp
2011‐11‐21/05–10 UT	263 nT	07:21 UT	1	06‐09 UT
2012‐01‐21/05–08 UT	290 nT	07:46 UT	2.33	03‐06 UT
2012‐03‐01/05–08 UT	565 nT	05:28 UT	3.67	06‐09 UT
2012‐03‐21/08–10 UT	365 nT	09:43 UT	1	06‐12 UT
2012‐03‐27/06–08 UT	236 nT	06:31 UT	1.67	06‐09 UT
2012‐04‐11/08–11 UT	229 nT	09:57 UT	2	06‐09 UT
2012‐11‐20/05–10 UT	373 nT	06:12 UT	2.67	03‐09 UT
2012‐12‐02/08–10 UT	815 nT	08:53 UT	2.67	06‐12 UT
2012‐12‐17/05–07 UT	444 nT	05:50 UT	2.67	03‐09 UT
2013‐03‐16/08–11 UT	603 nT	08:24 UT	2.67	06‐09 UT
2013‐03‐20/06–08 UT	364 nT	06:12 UT	1.67	06‐09 UT
2013‐04‐01/07–09 UT	428 nT	08:26 UT	1.67	06‐09 UT
2013‐04‐06/07–09 UT	228 nT	07:14 UT	1.67	06‐09 UT
2013‐04‐14/08–11 UT	299 nT	09:00 UT	3.33	06‐09 UT

*Note.* Also listed are the maximum AE and Kp index, and the corresponding times, during these intervals.

## 3 Case Study

### 3.1 Event 1 March 2012

**Figure 1:**
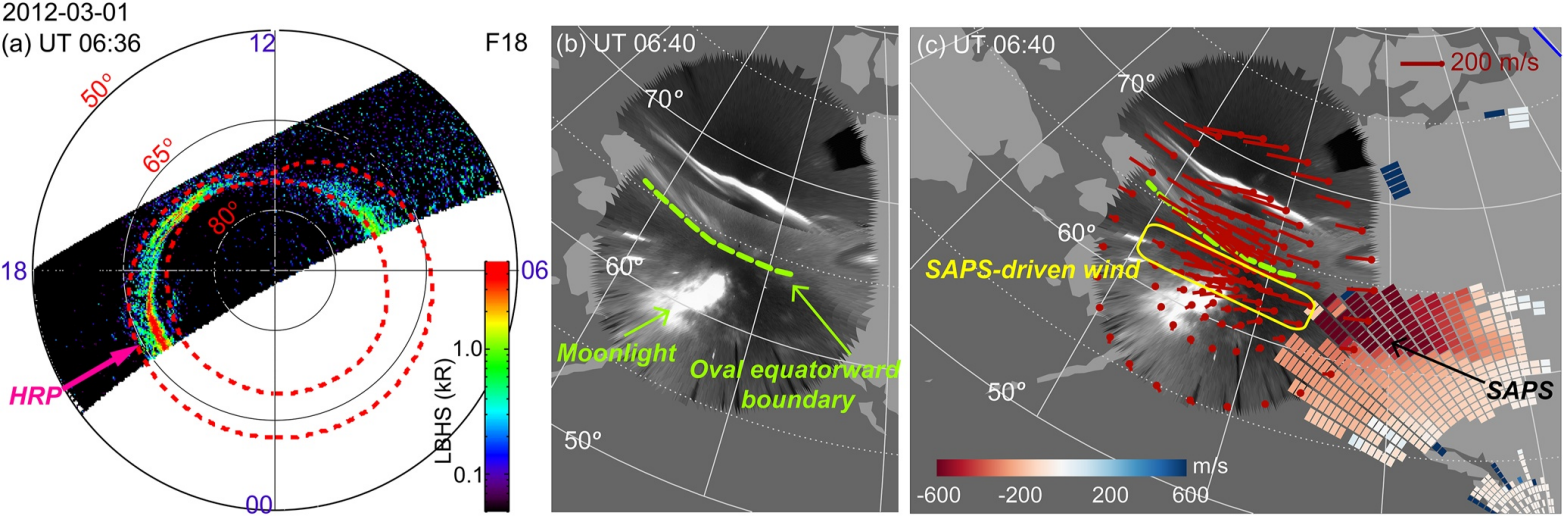
(a) DMSP F18 SSUSI observations of northern hemisphere auroras in the LBHS wavelength. The auroras are displayed in the MLAT‐MLT coordinates. Noon is to the top. The red dotted lines are the auroral boundaries provided by DMSP. The magenta arrow marks the location of the HRP SDI station. (b) THEMIS ASI observations of auroras at the Alaska region in white‐light emission in AACGM coordinates. The green dashed curve marks the equatorward boundary of the oval. (c) Neutral wind (red arrows) and plasma flow (color pixels) overlain on the THEMIS mosaic. The red arrows represent the horizontal velocity vectors of neutral wind (extending from each dot). The arrow directions give the velocity direction, and the arrow length gives the velocity magnitude. The color pixels represent the radar LOS data (negative means plasma flow away from the radar). Because CVW radar looks toward the west, SAPS appears as deep red color.

The event occurring during 0500–0800 UT on 1 March 2012 exemplifies situations when SAPS drives significant upper thermospheric winds. The maximum AE index during the interval of interest was 565 nT and Kp was 3.67. Figure 1a displays an auroral image from DMSP SSUSI at LHBS wavelength. The overlain red dotted contour lines mark the equatorward and poleward boundaries of the auroral oval. Figure 1b presents the THEMIS ASI mosaic in gray scale. Here, the equatorward boundary of the oval is visually identified as the boundary of the gray‐colored diffuse emissions, beyond which no other auroral emissions occurred (the bright spot at 59–63° MLAT was due to moonlight). This boundary is outlined as the green dashed curve. Both Figures 1a and 1b suggest that the equatorward boundary was located at ∼64.5° MLAT, and therefore plasma flows and neutral winds that occurred equatorward of 64.5° MLAT represent subauroral phenomena.

Figure 1c overplots the LOS measurements of the SuperDARN CVW radar on the THEMIS ASI mosaic. SAPS appears as red color tiles equatorward of the equatorward boundary of the oval, and one such channel occurred at 61°–65° MLAT. The winds, as measured by the HRP SDI, are denoted by the red arrows whose length is proportional to the wind speed. Westward winds occurred throughout the auroral oval, and rather than subsiding, they extended further equatorward to ∼62° MLAT. The westward direction is consistent with what is expected for the ion drag in the oval and the SAPS channel. The small meridional wind component suggests that the subauroral winds were not a result of equatorward advection from the auroral oval to the subauroral latitudes, but forced by SAPS. This is consistent with Ferdousi et al. (2019) who showed that the westward subauroral winds are predominantly forced by local momentum exchange between neutrals and ions (i.e., ion drag). Therefore, in this event, SAPS was associated with westward winds that merged with the winds in the auroral oval. Equatorward of the SAPS channel, the wind speed was small, and the wind direction turned from westward to eastward, consistent with a vanishing ion drag there and a dominating role of the pressure gradient produced by the dayside solar heating.

**Figure 2:**
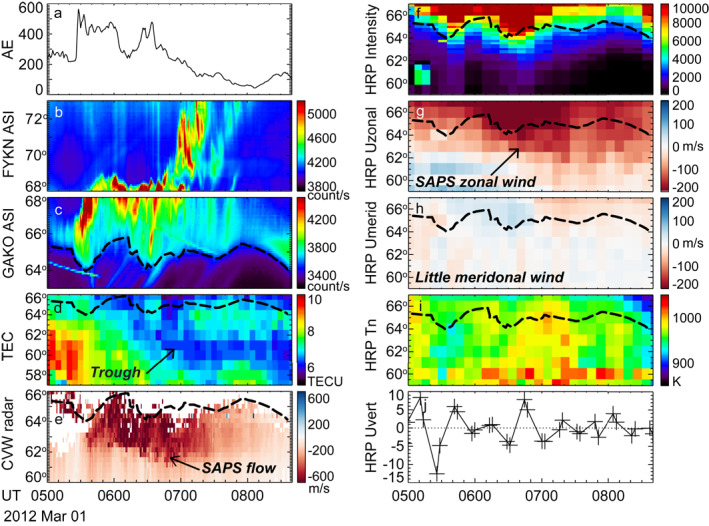
(a‐e) AE index from OMNI dataset, white‐light auroras measured by THEMIS Fort Yukon (FYKN) and Gakona (GAKO) stations, TEC measurements over the Alaska region, and LOS velocity measured by the SuperDARN CVW radar. The data are presented in AACGM coordinate system (Shepherd, 2014). FYKN is located at 66.6°N, −145.2°E in geographic, and 67.2°N and −94.8°E in geomagnetic coordinates. GAKO is located at 62.4°N, −145.2°E in geographic, and 63.1°N and −91.8°E in geomagnetic coordinates. The LOS velocity has been averaged cross Beams 12–15 and the red color implies plasma moving away from the radar. Because the radar looks to the west, the red color means plasma moving westward, which further corresponds to the sunward direction in the dusk to midnight sector. The black dashed curve in Figure 2c represents the equatorward boundary of the auroral oval and is also overlain on other panels. (f–j) 630.0 nm emission intensity, zonal winds, meridional winds, neutral temperature, and detrended vertical winds all measured by the HRP SDI.

Figure 2 presents the event in time series plots. Figure 2a shows that SAPS occurred over Alaska after a sharp increase of AE to 565 nT. An examination of Figures S1a and S1b in Supporting Information S1 further reveals that SAPS first developed to the west of Alaska over a limited local time sector (Panel a), and then extended across Alaska (Panel b) toward the magnetic midnight. Figures 2b and 2c show the auroral activity at Alaska measured by THEMIS ASIs, and the equatorward boundary of the oval is marked with the black dashed curve in Figure 2c. Equatorward of this boundary, the intensity of auroral emissions dropped to the background level. This boundary has been overlain on the TEC (Figure 2d), SuperDARN CVW radar (Figure 2e), and wind measurements (Figures 2f–2i). Note that the 630.0‐nm emission intensity detected by the HRP SDI can also be used to infer the location of the boundary, although the spatial and temporal resolution of the SDI is much lower than the THEMIS ASI. In Figure 2f, the ASI‐determined boundary follows the gradient of the SDI emission intensity with an error of ∼1° in latitude, the error being reasonable considering the SDI spatial resolution and the mapping uncertainty of the emissions (the white light emissions in THEMIS ASIs are mapped to 110 km, whereas the 630.0‐nm emissions in SDIs are mapped to 250 km). Data from the Poker Flat (PKF) SDI were also available and support the HRP SDI measurements (see Figure S2 in Supporting Information S1).

The SAPS flow and subauroral zonal winds seen in Figure 1 are clearly visible in Figures 2e and 2g. In Figure 2e, the LOS velocity was as large as ∼600 m/s until ∼0730 UT, after which the velocity gradually subsided to ∼300 m/s and below. In Figure 2g, the winds at subauroral latitudes had a speed ∼100 m/s during 0500–0600 UT, and increased to ∼150 m/s in the following hours. The smaller wind speed during 0500–0600 UT can be attributed to the fact that it takes time for winds to be accelerated to a large speed due to the large inertial of neutrals. It may also reflect the fact that at this time, the SDI was measuring winds at dusk, where the pressure gradient force is strong and opposes the ion drag associated with SAPS. Although SAPS weakened considerably after 0730 UT, winds persisted with only a modest decrease, again possibly due to the combination of the large inertia of the neutrals and still some weakened SAPS forcing. Studies have suggested that winds can persist up to 6 hr after the forcing cases (Lyons et al., 1985). By focusing on winds of >50 m/s, the equatorward edge of the zonal winds was located at ∼62° MLAT for most of the time of our interest, similar to that of SAPS. According to the GPS TEC data in Figure 2d, the SAPS was located at the poleward portion of the midlatitude trough (note that the y range of Figure 2d is much larger than Figure 2e) where TEC was reduced by ∼20% compared with the surrounding.

Figures 2h and 2i show that no substantial meridional wind or enhanced temperature is observed in association with the SAPS, which is different from previous observations and simulations (H. Wang et al., 2011; Wang, Lühr, et al., 2012; Wang, Talaat, et al., 2012; Zhang et al., 2015). This is found to be common in our database (see Section 4), and the difference may be because during non‐storm time SAPS and its forcing on the upper thermosphere are weak. In comparison, the poleward wind surge in Zhang et al. (2015) was associated with a storm‐time wind of ∼300 m/s, the speed being twice as large as this event. The temperature enhancement in Wang, Talaat, et al., 2012 was confined to regions where the SAPS and wind speeds exceeded ∼500 and ∼200 m/s, respectively.

Guo et al. (2018) identified acoustic‐gravity waves associated with SAPS based on perturbations in the vertical winds, and Figure 2j shows the detrended vertical wind. The detrending is performed with a 90‐min moving average. Guo et al. (2018) proposed that the SAPS induced acoustic‐gravity waves have a period of 10–11 min and can last >1 h. The amplitude increases with altitude, reaching ∼200 m/s at ∼500 km (Figure 5 in Guo et al., 2018). However, Figures 2j and S2j in Supporting Information S1 only showed small amplitude fluctuations (<10 m/s). Although the exact source is not known, similar fluctuations also occurred when SAPS failed to drive substantial thermospheric winds (Sections 3.2 and 3.3) and hence they may not be driven by SAPS but by other processes. The absence of the waves may again be due to the weak Joule heating in this event because the simulation in Guo et al. (2018) was for a storm with a Sym‐H < −200 nT. Nygrén et al. (2015) proposed that one possible source of the weak fluctuations is the terminator.

### 3.2 Event 27 March 2012

**Figure 3:**
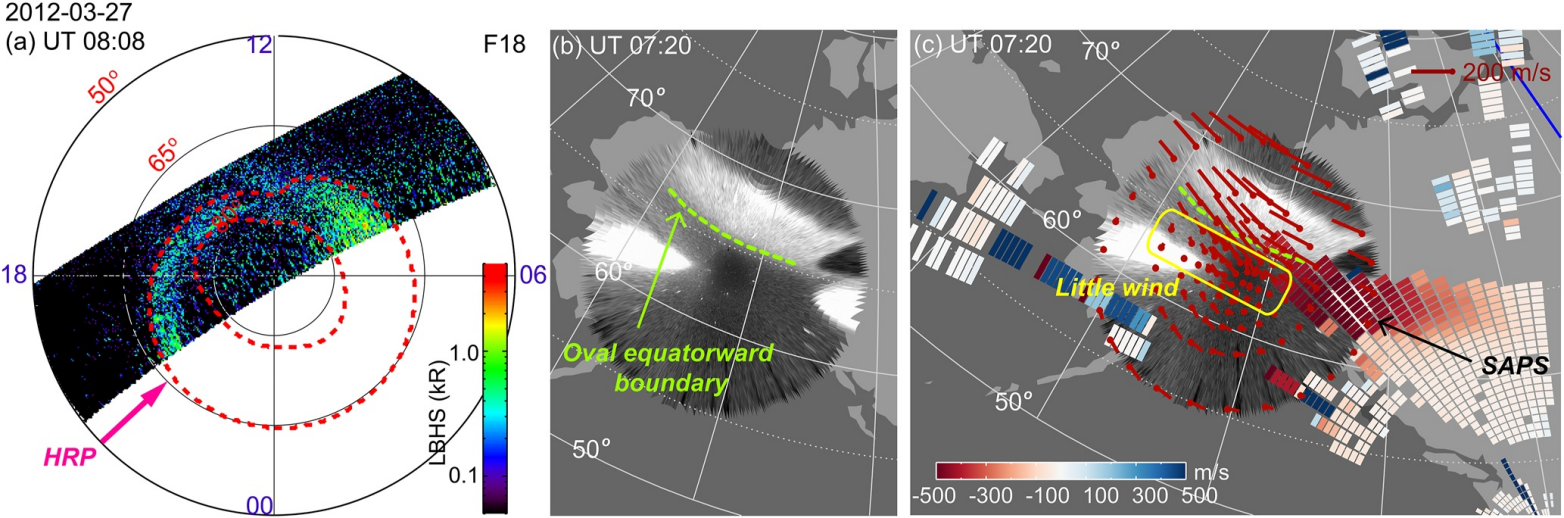
Similar to Figure 1 but for Event 27 March 2012.

**Figure 4:**
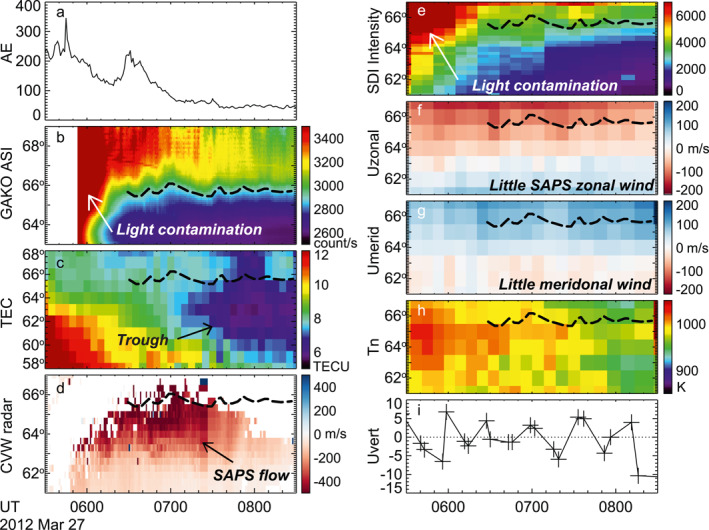
Similar to Figure 2 but for Event 27 March 2012. The LOS velocity in Figure 4d is obtained from Beams 13‐16 from the CVW radar.

Different from the event above, the SAPS during 0600–0800 UT on 27 March 2012 did not produce significant effects on the upper thermosphere. This event had a maximum AE of 236 nT and Kp of 1.67. Figures 3a and 3b display auroras measured by DMSP SSUSI and THEMIS ASI. The two images are similar despite being taken at different times because the auroral activity was quasi‐steady (see Figure 4 discussed below). Both instruments suggest that the equatorward boundary of the auroral oval was located at ∼65.5° MLAT. Equatorward of the oval, a SAPS channel occurred at ∼62.5°–65.5° MLAT, as seen from the SuperDARN CVW measurements in Figure 3c. Interestingly, this SAPS was not associated with westward winds. The westward winds occurred primarily within the auroral oval, and subsided quickly equatorward of the oval. For example, at and equatorward of 64° MLAT, the winds were weak and turned from westward to eastward.

The absence of SAPS‐associated winds is also evident from Figure 4. Figure 4a shows that the event occurred as AE decreased from a peak value of 346 nT at 0545 UT, and therefore the geomagnetic activity was overall lower than the first event. Figure 4b shows that the equatorward boundary of the auroral oval oscillated around ∼65.5° MLAT during 0630–0830 UT. This is supported by Figure 4e, where the SDI‐measured emission intensity dropped rapidly below 65°. Figure 4d shows that a SAPS channel occurred between this boundary and 62.5° MLAT. The SAPS had a peak velocity of ∼500 m/s and the velocity decreased after 0730 UT. After 0800 UT, the SAPS nearly disappeared. According to the TEC measurements in Figure 4c, the midlatitude trough did not extend to the Alaska region until ∼0700 UT, and after that SAPS was positioned at the poleward portion of the trough.

Despite of the existence of the SAPS, Figure 4f shows that westward winds were mostly confined to the region at and above 65° MLAT. Equatorward of 63° MLAT, the wind turned to eastward. Although SAPS may have still exerted forcing on the thermosphere at 64° MLAT (see the light red color where the zonal speed fluctuated about 30–40 m/s), the fact that the winds were significantly weaker than those in Figures 1 and 2 indicates a weak effect of SAPS.

We did not observe clear meridional winds (Figure 4g) or temperature enhancement (Figure 4h) in association with the SAPS, implying a weak Joule heating. Joule heating has been shown to correlate with the AE index (e.g. Robinson & Zanetti, 2021), and the AE of this event is even smaller than that of the first event. The fluctuations in the vertical velocity were small (<10 m/s; Figures 4h–4i), similar to the first event.

### 3.3 Event 20 November 2012

**Figure 5:**
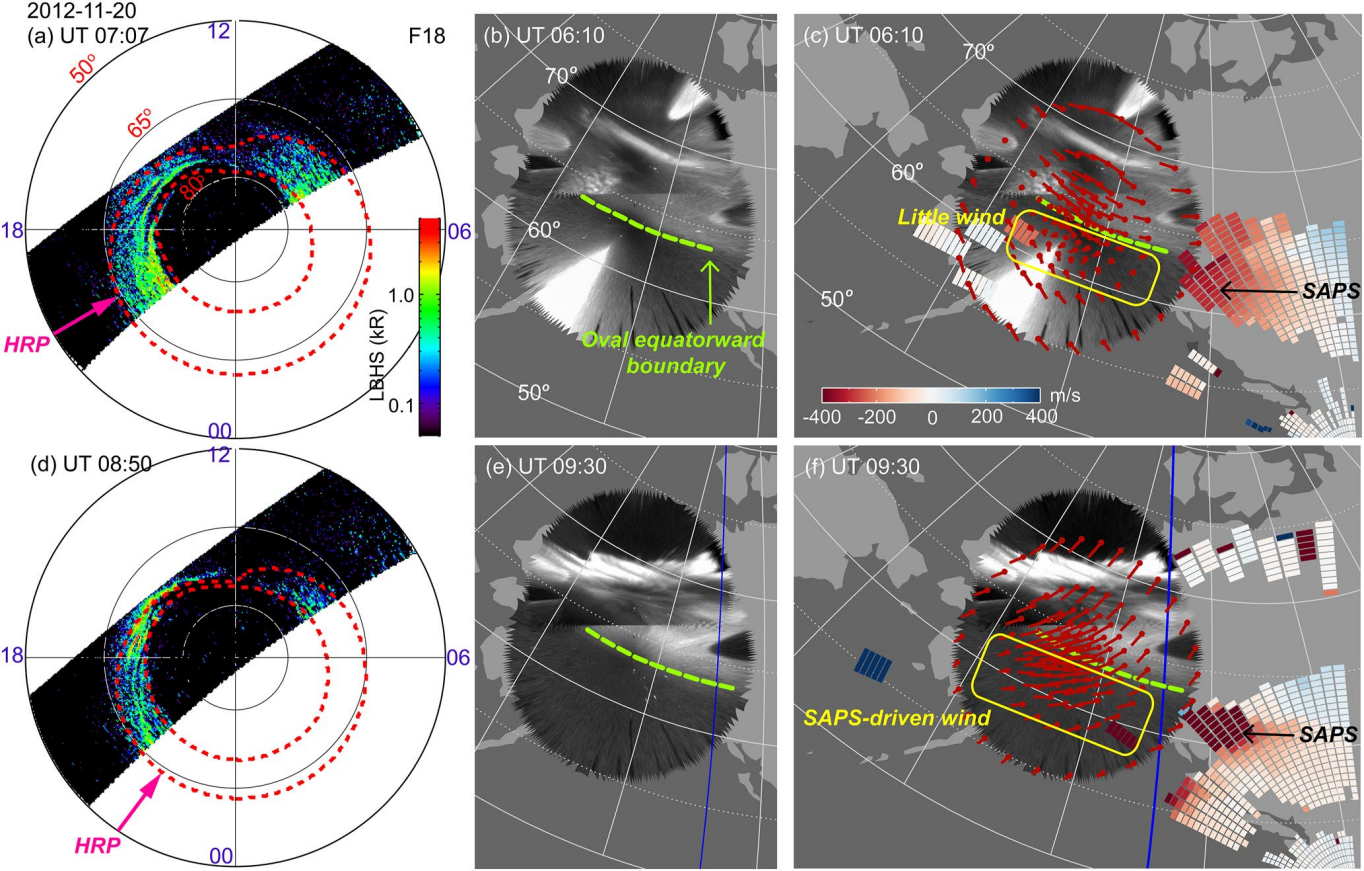
Similar to Figure 1 but for Event 20 November 2012. The top row corresponds to a time when no westward neutral winds occurred at subauroral latitudes, and the bottom to a time when the winds occurred.

The event occurring during 0500–1000 UT on 20 November 2012 serves as an example when SAPS drives upper thermospheric winds during a portion of its duration, whereas little winds during the rest. The event had a maximum AE of 373 nT and Kp of 2.67. Figure 5 shows SAPS and the associated wind field at the dusk sector (top row) and before magnetic midnight (midnight at ∼1100 UT, bottom row). Figures 5a and 5b present that at dusk, the equatorward boundary of the auroral oval was located at ∼64° MLAT. Equatorward of this boundary plasma flows were directed westward down to ∼60° MLAT with a velocity of ∼300 m/s, as shown in Figure 5c, suggesting the presence of SAPS. This SAPS, however, was not associated with westward winds. Instead, westward winds were limited to the auroral oval, i.e., the region at >64° MLAT. The winds equatorward of the oval were either very small (<30 m/s) or were directed eastward.

On the other hand, Figures 5d–5f show that before midnight, the equatorward boundary of the auroral oval was located at ∼63.5° MLAT and a SAPS channel occurred between this boundary and ∼60° MLAT. When examining the wind vectors, we find that the winds in the auroral oval had a significant southward component in addition to the westward component. The southward wind could arise from the ion drag in the oval because plasma flows are directed southward around midnight in the classic two‐cell convection pattern, as well as from the pressure gradient that is set up by auroral particle precipitation and Joule heating. Here the auroral particle precipitation is manifested as the suddenly brightened auroras that expanded poleward during 09–10 UT. The southward wind continued to the subauroral latitudes, where they gained a substantial westward component as expected from the ion drag of the SAPS.

**Figure 6:**
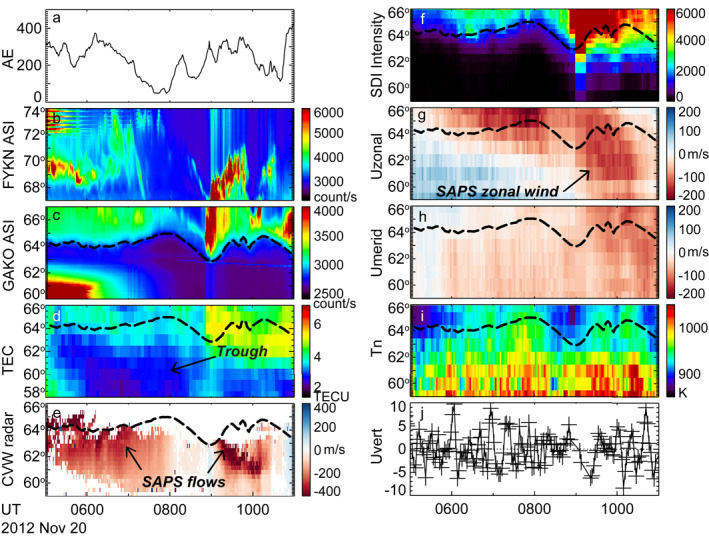
Similar to Figure 2 but for Event 20 November 2012.

The time dependent wind behavior can be seen from Figure 6. Figure 6g shows that during 0500–0700 UT, the westward winds occurred mostly above ∼64° MLAT despite that, according to Figure 6e, the SAPS extended to 60° MLAT beyond which limited backscatter was received by the radar. During 0700–0900 UT, the westward winds penetrated to subauroral latitudes, and acquired a velocity of ∼100 m/s. The latitudinal location of the equatorward edge of the wind decreased with time. After 0900 UT and until the end of the interval of our interest, the winds extended below ∼60° MLAT. This is also the time when the southward wind component in Figure 6h increased to >50 m/s.

The southwestward winds at subauroral latitudes have also been reported by Zou et al. (2021) and Liang et al. (2021). Zou et al. (2021) found that these winds are a common feature of substorms, and usually occur in the first hour following the substorm onset. Due to the lack of plasma flow measurements in that study, the authors could only postulate that the winds are driven by SAPS. The postulation was based on the fact that the winds were located at subauroral latitudes and directed westward, consistent with SAPS. Figure 5 confirms that SAPS indeed concurred with the southwestward winds. Liang et al. (2021) examined the impact of the southwestward winds on a subauroral process named STEVE (“Strong Thermal Emission Velocity Enhancement). In STEVE events, the winds feature a steep stop/reversal at the subauroral latitude, where strong wind convergence is developed. Such a reversal is absent in non‐STEVE events. The authors proposed that the southward component of the winds transports relevant neutrals species that are key to the STEVE airglow production from the auroral zone to subauroral latitudes, and leading them to pileup there. This results in a reservoir of neutral constituent which, when further aided by SAID, leads to a dramatic increase of the airglow production and the STEVE occurrence. The event under analysis did not exhibit a steep stop/reversal of the southward winds, and no STEVE occurred.

## 4 Statistical Analysis

### 4.1 Occurrence of SAPS‐Driven Winds

As indicated in Section 3, SAPS sometimes drives westward winds of >50 m/s at subauroral latitudes, and here we examine what controls the occurrence of SAPS‐related winds. As mentioned in Section 2, we have identified 14 events between October 2009 and May 2014 satisfying the event criteria. To properly characterize events like the one in Section 3.3, where SAPS drives upper thermospheric winds during only a portion of its duration, we separate each event into 1‐hr bins and determine the occurrence of SAPS‐related winds within each bin. SAPS‐related winds are defined as occurring when there exist westward winds with a speed >50 m/s at subauroral latitudes. There are in total 38 bins and 23 bins are associated with SAPS‐related winds. Our database is somewhat limited because of the short lifetime of HRP SDI and the requirement of simultaneously good measurements from several instruments. As a result, the statistics below can lack statistical significance under certain conditions and caution is needed in analyzing. Nevertheless, our primitive statistical results would provide helpful information for future observations, such as those to be made by Geospace Dynamics Constellation, as well as simulations.

**Figure 7:**
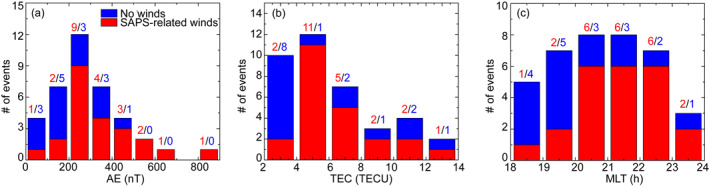
Histograms showing the number of events when westward neutral winds occurred at subauroral latitudes in association with SAPS (red) and when no winds occurred (blue). The histograms are shown as a function of AE index (Figure 7a), TEC (Figure 7b), and magnetic local time (Figure 7c). Magnetic midnight at the HRP SDI is ∼11 UT. The numbers in red (blue) on top of each bar represents the number of SAPS‐related wind events (no wind events) within that bar.

Figure 7 shows the dependence of the wind occurrence on AE index, local time, and TEC. As discussed in the introduction, the latitudinal location, the longitudinal extent, and the peak velocity of SAPS all vary with geomagnetic activity, indicating that the winds associated with SAPS may also vary with geomagnetic activity. Here the geomagnetic activity is characterized by the peak AE value within each 1‐hr bin. Figure 7a shows that the occurrence of SAPS‐related westward winds is 27% (3 out of 11 events) when AE < 200 nT, and 68% (13 out of 19 events) when 200 nT < AE < 400 nT. While events at AE > 400 nT are limited in number, they are consistent with the trend of increasing wind occurrence with increasing AE (7 out of 8 events).

Considering that ion drag is one of the most important forces in driving thermospheric winds, the wind occurrence may potentially vary with the collision frequency between the neutrals and the ions. The frequency is proportional to the density of ions, and here we use TEC as a proxy because direct density measurements are not available. We take the TEC data within 1 hr local time from the SDI station and within the SAPS channel, and compute the median value within each 1‐hr bin. Figure 7b shows that SAPS‐related winds can occur for a wide range of TEC but the occurrence rate is smaller when TEC <4 TECU (2 out of 10 events). Events at TEC >8 TECU are limited in number, but they imply that a high TEC does not necessarily result in a high wind occurrence because the overall rate is merely 56% there (5 out of 9 events). This finding indicates that although TEC can affect the SAPS effect on winds, it may not be a dominant factor when it increases above a certain value (which is 4 TECU according to Figure 7b).

The wind occurrence may also vary with local time, because the ion drag is opposed by the pressure gradient force associated with dayside solar heating, and the gradient of the heating is larger at dusk than the nightside sector. This hypothesis is supported by Figure 7c, where the occurrence is 25% (3 out of 12 events) at 18–20 h MLT, and increases to 78% (18 out of 23 events) at 20–23 h MLT. The occurrence is still high at 23–24 h MLT although the number of events at this MLT is small and may lack statistical significance. The dependence on local time may explain why, for the event in Section 3.3, the subauroral winds were absent before 20 h MLT despite the presence of SAPS.

### 4.2 Location of SAPS‐Related Winds

As reviewed in the introduction, the latitude of SAPS decreases with increasing geomagnetic activity and MLT. Here we examine the location of the equatorward edge of the SAPS‐related winds as a function of AE index and MLT, and compare with the edge of the SAPS channels. To determine the equatorward edge of SAPS‐related winds, we identify the latitude where the velocity drops to <50 m/s at a spatial resolution of 1°. If the winds extend equatorward of the SDI FOV at 58°, the edge is marked as 58°. On the other hand, to determine the equatorward edge of SAPS, good radar backscatter over a broad latitude range is needed, and 17 out of the 23 SAPS‐related winds satisfy this requirement. The velocity threshold for SAPS equatorward edge is set as 200 m/s. One limitation of the following analysis is that the number of events with large AE (AE > 400 nT) is limited, and we don't have events occurring after 24 h MLT because SAPS has a low occurrence there (e.g., Foster & Vo, 2002).

**Figure 8:**
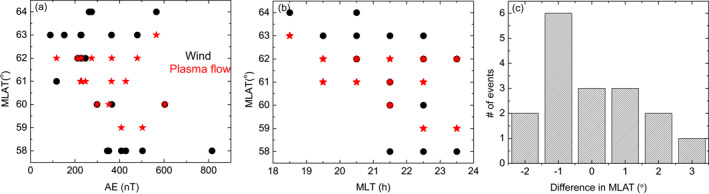
(a–b) Dependence of the equatorward boundary of the subauroral winds (black solid dots) and SAPS (red stars) on AE index and magnetic local time. (c) Difference in latitudes between the equatorward edges of SAPS and SAPS‐related winds. Positive means that winds extend equatorward of SAPS.

As shown in Figure 8, the winds extend to low latitudes, for example, 60° MLAT and below, at large AE and around midnight. This trend is consistent with SAPS. The MLT dependence has considerable data scatter, where SAPS and associated winds still occurred at >61° in the MLT range of 23–24 h. A careful check of these data points reveals that they occurred during small AE of 89, 212 and 229 nT.

Figure 8c shows the difference in latitudes between the equatorward edges of SAPS and SAPS‐related winds when both edges can be determined simultaneously. The wind is roughly collocated with the SAPS with a difference varying within −2°−3°. Here positive means that winds extend equatorward of SAPS. The difference shows a peak at −1°, which is the case in Figures 1c and 2g. One example of winds extending equatorward of SAPS can be found in Figures 5f and 6g where the winds reached to 59° MLAT during 0900–1000 UT whereas the SAPS stopped at 61° MLAT. The forcing of more equatorward winds warrants further study, but we postulate it to be related to advection associated with the southward winds in the oval.

### 4.3 Speed of SAPS‐Related Winds

**Figure 9:**
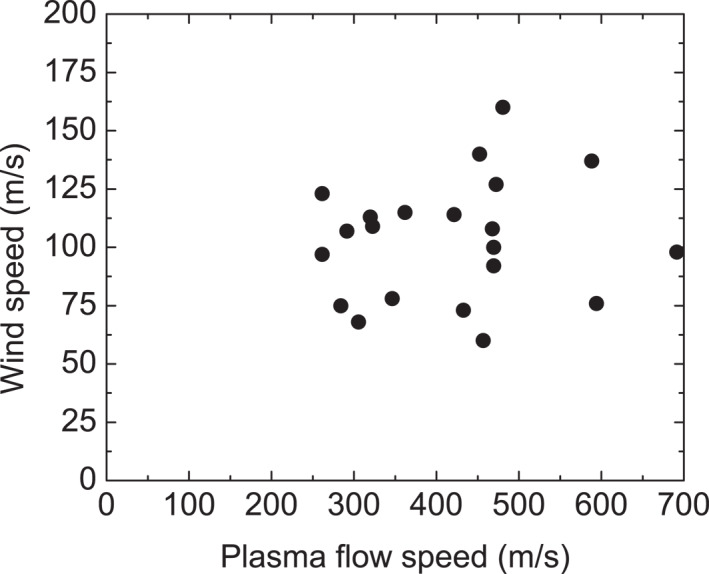
Dependence of the zonal speed of the subauroral winds on SAPS speed.

Figure 9 shows the zonal wind speed as a function of the plasma flow velocity. Here we assume that SAPS velocity is nearly zonal, and therefore the line‐of‐sight measurements are corrected with a magnetic direction cosine factor to yield the zone flow component (Erickson et al., 2011). A majority of the SAPS channels has a speed between 300 and 500 m/s, comparable to the statistical result in Kunduri et al. (2017, 2018) where, for non‐storm time intervals, a median speed in the range of 300–400 m/s was obtained.

The winds usually have a zonal speed of 60–160 m/s. To obtain the ratio between the wind and plasma flow speed, we conduct a linear fitting by assuming the intercept to be zero (i.e., wind speed being zero when the plasma flow is stagnant) and find the slope to be 0.21. This number is smaller than H. Wang et al. (2011), where they found that the SAPS‐related winds have a velocity about 35% of the plasma velocity. The result implies a weaker ion‐neutral coupling during non‐storm than active time. Note that the ratio of 0.21 is similar to those in Zou et al. (2018) studying meso‐scale winds around auroral forms.

## 5 Summary

The effect of non‐storm SAPS on the upper thermosphere has been investigated based on simultaneous observations made by the HRP SDI, SuperDARN radars, THEMIS ASIs, DMSP spacecraft, and GPS TEC measurements. The SAPS under analysis corresponds to enhanced flows that are 2°–5° wide equatorward of the auroral oval. The results represent the ion‐neutral coupling at subauroral latitudes during non‐storm time. Our findings are summarized as follows.

1. SAPS at times drives substantial (>50‐m/s) westward winds at subauroral latitudes in the dusk‐midnight sector, but not always. The occurrence of the westward winds varies with AE index, plasma content in the trough, and the local time, where large AE, modestly large TEC, and the nightside sector favor the wind formation. The dependence on the local time is probably because the ion drag associated with the SAPS flows is opposed by the pressure gradient associated with dayside solar heating, and this gradient is larger at dusk than the nightside sector
2. The winds usually have a speed of 60–160 m/s. The zonal wind speed averages 21% of the zonal plasma speed
3. The SAPS‐related winds extend to low latitudes, for example, 60° MLAT and below, at large AE and around midnight. This trend is consistent with SAPS. The wind tends to have an equatorward boundary that is 1° poleward of SAPS (given a spatial resolution of 1°), although they may extend equatorward of SAPS around midnight possibly due to advection associated with the southward winds in the oval
4. SAPS is not observed to drive poleward wind surge, neutral temperature enhancement, or acoustic‐gravity waves, likely due to the somewhat weak SAPS forcing during the non‐storm time we focus on

The occasional absence of SAPS‐related winds is not found in the statistical analysis of H. Wang et al. (2011), possibly due to two reasons. First, as mentioned in the introduction, the SAPS in H. Wang et al. (2011) is strong and wide, whereas our SAPS has the typical a speed and width expected for non‐storm time. Second, the wind measurements in H. Wang et al. (2011) were made at a higher altitude (400 km) than ours (250 km). Whether and how the SAPS‐relate winds vary with altitude warrants future study.

## Supporting information

Supporting Information S1

## Data Availability

DMSP SSUSI data are available through https://ssusi.jhuapl.edu/data_products. GPS TEC data products and access through the Madrigal distributed data system (http://isr.sri.com/madrigal/) are provided to the community by the Massachusetts Institute of Technology under support from U.S. National Science Foundation grant AGS‐1242204. Data for the TEC processing are provided from the following organizations: UNAVCO; Scripps Orbit and Permanent Array Center; Institut Géographique National, France; International GNSS Service; the Crustal Dynamics Data Information System (CDDIS); National Geodetic Survey; Instituto Brasileiro de Geografia e Estatística; RAMSAC CORS of Instituto Geográfico Nacional de la República Argentina; Arecibo Observatory; Low‐Latitude Ionospheric Sensor Network (LISN); Topcon Positioning Systems, Inc.; Canadian High Arctic Ionospheric Network; Institute of Geology and Geophysics, Chinese Academy of Sciences, China Meteorology Administration; Centro di Ricerche Sismologiche; Système d'Observation du Niveau des Eaux Littorales (SONEL); RENAG: REseau NAtional GPS permanent; GeoNet New Zealand; and GNSS Reference Networks. The authors thank Vassilis Angelopoulos for the use of THEMIS data.
