# Supplementary material for: Effects of Subauroral Polarization Streams on the Upper Thermospheric Winds During Non‐Storm Time
Source: J Geophys Res Space Phys. 2022 Apr 27;127(5):e2021JA029988. doi: 10.1029/2021JA029988 (PMC9286583; doi:10.1029/2021JA029988)
Supplement: Supplementary file 1 — Supporting Information S1 [file JGRA-127-0-s001.docx]

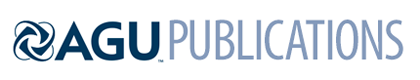


[Journal of Geophysical Research: Space Physics](https://agupubs.onlinelibrary.wiley.com/journal/21699402)

Supporting Information for

**Effects of Subauroral Polarization Streams on the Upper Thermospheric Winds during Non-Storm Time**

Ying Zou1, Larry Lyons2, Xueling Shi3,4, Jiang Liu2,5, Qian Wu4, Mark Conde6, Simon G. Shepherd7, Stephen Mende8, Yongliang Zhang9, Antea Coster10

1. Department of Space Science, University of Alabama in Huntsville, USA

2. Department of Atmospheric and Oceanic Sciences, University of California, Los Angeles, California, USA

3. The Bradley Department of Electrical and Computer Engineering, Virginia Tech, Blacksburg, Virginia, USA

4. High Altitude Observatory, National Center for Atmospheric Research, Boulder, CO, USA.

5. Department of Earth, Planetary and Space Sciences, University of California, Los Angeles, California, USA

6. Department of Physics, University of Alaska Fairbanks, Fairbanks, Alaska, USA

7. Thayer School of Engineering, Dartmouth College, Hanover, NH, USA

8. Space Sciences Laboratory, University of California, Berkeley, California, USA

9. Applied Physics Laboratory, Laurel, Maryland, USA

10. Massachusetts Institute of Technology Haystack Observatory, Westford, Massachusetts, USA

**Contents of this file**

Figures S1, S2

**Introduction**

Figure S1 shows the global convection maps made by SuperDARN. These measurements are consistent with, and hence support, the LOS velocity data presented in the paper.

Figure S2 shows the upper thermosphere measurements made by the Poker Flat (PKR) Scanning Doppler Imager (SDI). These measurements are consistent with, and hence support, the HRP SDI data presented in the paper.


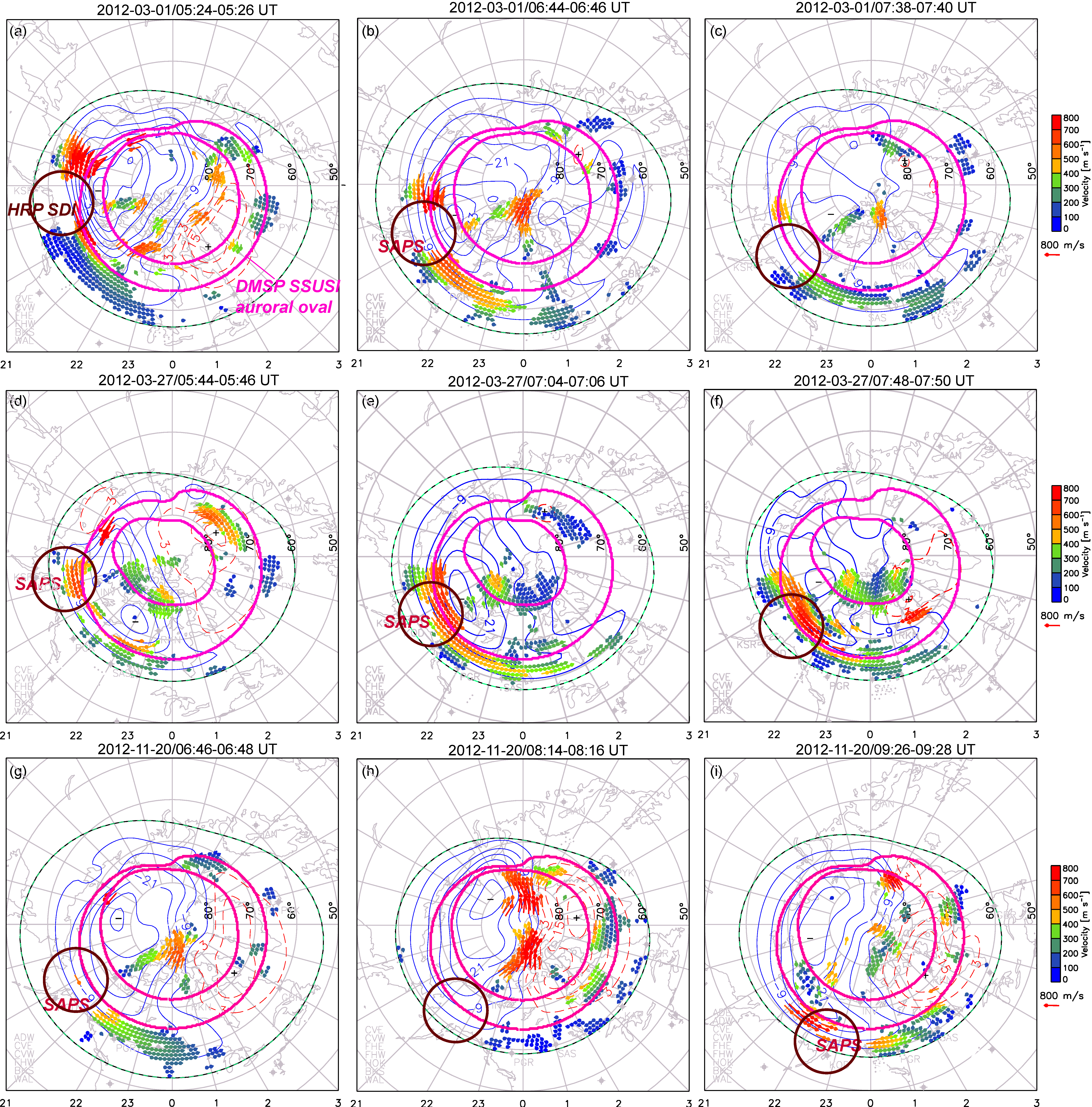


Figure S1. Global convection maps showing plasma convection vectors at representative instances. The colored arrows represent the fitted velocity vectors (extending from each dot). The first, second, and third row corresponds to the first, second, and third case study, respectively. DMSP SSUSI auroral boundaries are overlain as magenta contours. The FOV of the HRP SDI is marked with the maroon circle. The label of “SAPS” suggests that SAPS occurred over Alaska.


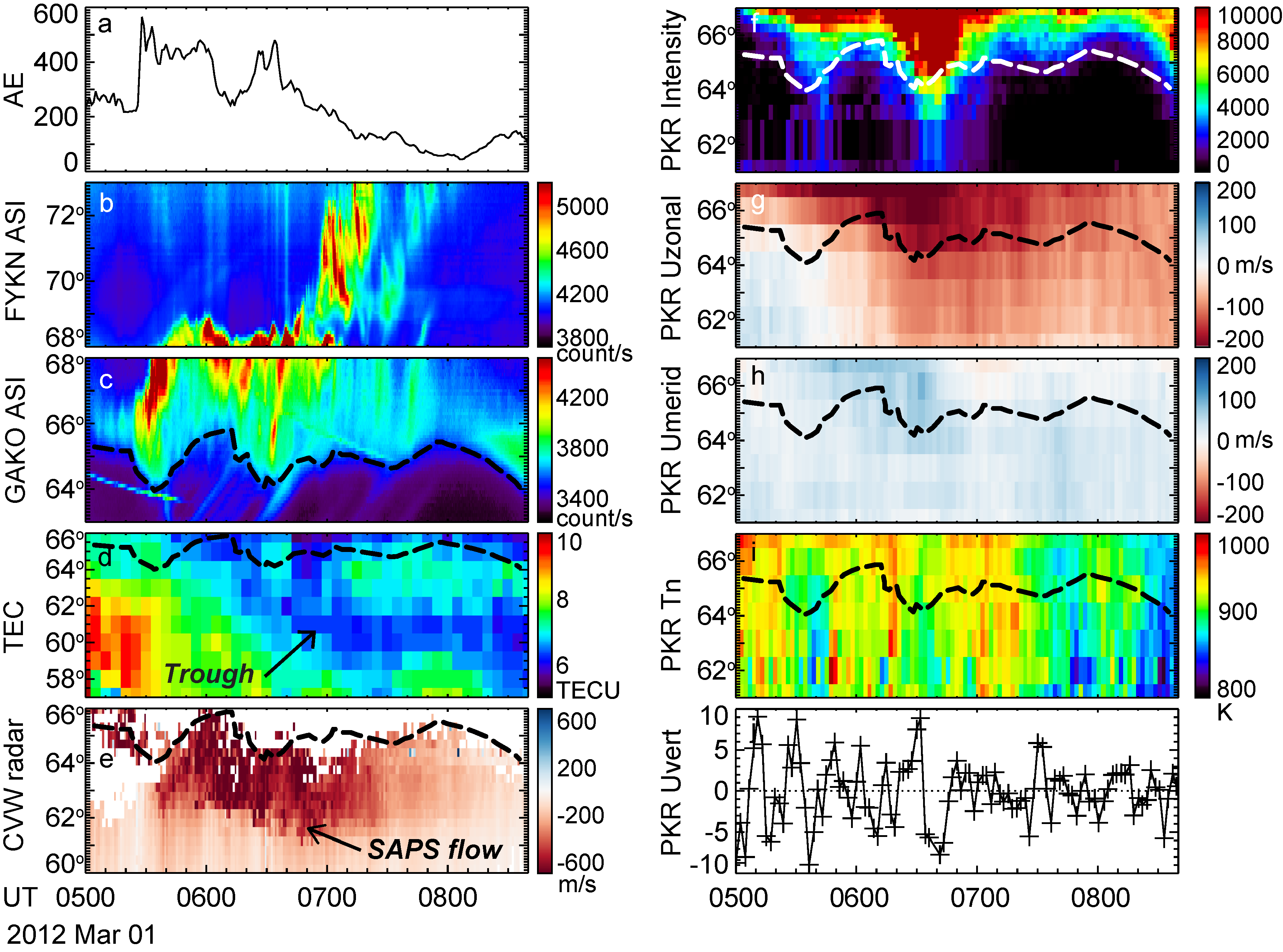
 Figure S2. (a-e) AE index from OMNI dataset, white-light auroras measured by THEMIS FYKN and GAKO stations (both located at the Alaska region), TEC measurements over the Alaska region, and LOS velocity measured by the SuperDARN CVW radar. The LOS velocity has been averaged cross Beams 12-15 and the red color implies plasma moving away from the radar. The black dashed curve in Figure S1c represents the equatorward boundary of the auroral oval and is also overlain on other panels. (f-j) 630.0 nm emission intensity, zonal winds, meridional winds, neutral temperature, and detrended vertical winds all measured by the PKR SDI.
